# High CEA levels in a case of resected colorectal cancer: delayed diagnosis of metachronous medullary thyroid cancer

**DOI:** 10.1186/s12957-017-1303-4

**Published:** 2017-12-29

**Authors:** Shih-Wei Chen, Yen-Kung Chen

**Affiliations:** 1 Department of Nuclear Medicine, Shin Kong Wu Ho Su Memorial Hospital, No. 95, Wen-Chang Rd., Shih-Lin District, Taipei, 11101 Taiwan; 2 School of Medicine, Taipei Medical University and Fu Jen Catholic University, New Taipei City, Taiwan; 3 Department of Nuclear Medicine and PET Center, Shin Kong Wu Ho-Su Memorial Hospital, No. 95, Wen-Chang Rd., Shih-Lin District, Taipei, Taiwan

**Keywords:** Carcinoembryonic antigen (CEA), Colorectal cancer, Fluorodeoxyglucose-positron emission tomography (FDG-PET), Medullary thyroid cancer, Calcitonin

## Abstract

**Background:**

Carcinoembryonic antigen (CEA) is one of the most widely used tumor markers, and its value in the surveillance of post-operative colorectal cancer is well established. Fluorodeoxyglucose-positron emission tomography (FDG-PET) has been clinically used in colorectal cancer imaging including preoperative staging, evaluation of therapeutic response, detection of disease recurrence, and investigation of unexplained rising tumor markers.

**Case presentation:**

We report a case of resected colorectal cancer presented with rising CEA levels in 5 years, and FDG-PET revealed no definitive evidence of recurrence except abnormal focal FDG uptake in the right thyroid lobe. However, fine needle aspiration cytology (FNAC) of the thyroid nodule showed negative for malignancy. Progressively rising CEA levels were noted over the following 5 years, but serial follow-up examinations did not find evidence of recurrence. Fluorodeoxyglucose-positron emission tomography/computed tomography (FDG-PET/CT) was performed subsequently and again showed focal FDG uptake in the right thyroid lobe. This time, FNAC revealed positive for malignancy, in favor of medullary thyroid carcinoma (MTC). The patient underwent total thyroidectomy and modified radical neck dissection, and MTC with cervical nodal metastasis (pT3N1) was diagnosed. He had cervical lymph nodes recurrence 2 years later, which was resected.

**Conclusions:**

This case reminded us that FDG-PET/CT may detect occult tumors resulting in CEA elevation other than colorectal cancer. Moreover, FNA has a higher false negative rate in detecting MTC than other forms of thyroid cancer. Repeat FNAC for the initial negative cytology result and measure of serum calcitonin for the early MTC detection could be more helpful to avoid the delay in MTC diagnosis.

## Background

Carcinoembryonic antigen (CEA) is one of the most widely used tumor markers, and its value in the surveillance of post-operative colorectal cancer is well established [1, 2]. However, it is also overexpressed in many different tumors, such as lung cancer and neuroendocrine pancreatic tumor. Fluorodeoxyglucose-positron emission tomography (FDG-PET) has several well-recognized applications in colorectal cancer imaging including preoperative staging, evaluation of therapeutic response, detection of disease recurrence, and investigation of unexplained rising tumor markers [3, 4]. In addition, the whole body scan of FDG-PET may detect occult metachronous tumors.

## Case presentation

**Fig. 1:**
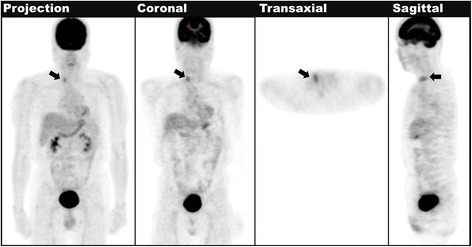
The first time FDG-PET scan of the patient of resected colorectal cancer with elevated CEA level (68 µg/L) showed focal FDG uptake in the right thyroid lobe (*arrow*)

A 56-year-old man with a history of colorectal cancer (CRC) status post curative resection presented with high serum CEA level up to 68 μg/L (normal limit < 5 μg/L, measured by radioimmunoassay) 5 years after surgery. To investigate for possibility of cancer recurrence, distant metastasis, or other tumors, we performed FDG-PET (Siemens Ecat Exact HR+ scanner). It demonstrated mildly focal increased FDG uptake in the right lobe of the thyroid gland, and maximum standardized uptake value (maxSUV) was 2.5 (Fig. 1). We did not observe other pathologic FDG uptake in the PET images. Therefore, the patient underwent ultrasound-guided fine needle aspiration (FNA) of the hypermetabolic thyroid nodule, and the cytology revealed negative for malignancy.

**Fig. 2:**
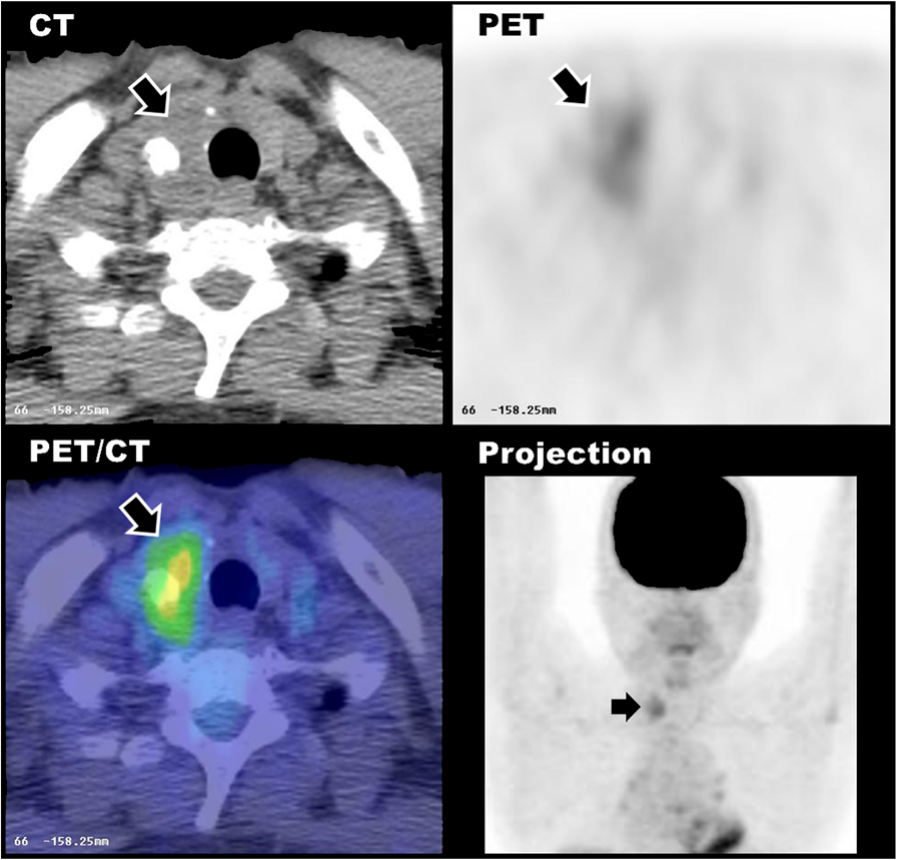
The second time FDG-PET/CT scan of the same patient with progressively elevated CEA levels up to 184 µg/L showed persistent focal FDG uptake in the right thyroid lobe (*arrow*), corresponding to the hypodense nodule and calcification shown on CT

Progressively rising CEA levels up to 184 μg/L were noted over the following 5 years. However, serial follow-up abdominal ultrasound, computed tomography (CT), and colonoscopy did not find evidence of recurrent disease of CRC. Subsequently, we performed FDG-PET/CT (GE Discovery LS PET/CT hybrid scanner) again and observed a hypermetabolic focus in the right thyroid lobe (maxSUV 3.5), which corresponded to the hypodense nodule and calcification shown on CT images (Fig. 2). This time, repeat FNA of the thyroid was done and revealed positive for malignancy, in favor of medullary thyroid cancer (MTC). The patient underwent bilateral total thyroidectomy and modified radical neck dissection. The histopathology revealed right thyroid medullary carcinoma with a tiny microscopic focus suggesting papillary carcinoma (pT3). Two right neck lymph nodes are metastasized by medullary carcinoma (pN1). The CEA level dropped to 30 μg/L two and a half months after surgery.

The subsequent follow-up showed fluctuating CEA levels (3.7 ~ 15.5 μg/L) and progressively rising calcitonin levels (up to 843 ng/L) in the first year to the second year after surgery. Ultrasound and FNAC revealed recurrent cervical lymphadenopathy. The patient received lymph nodes dissection and recurrent nodal metastasis from MTC which was proven. At this point, the patient continued regular follow-up in our hospital and the CEA and calcitonin levels remained within normal limits.

## Discussion

CEA is a glycoprotein present in normal mucosal cells, and its increased serum level is associated with adenocarcinoma, especially CRC. CEA level is useful in assessing prognosis, detecting recurrence, and monitoring treatment in patients with CRC [1, 2]. However, it may be elevated in a wide variety of other tumors and benign conditions, such as MTC, lung cancer, neuroendocrine pancreatic tumor, smoking, and infections. A recent systematic review has raised some doubts about the clinical usefulness of CEA in detecting recurrence after intended curative surgery for CRC, which included a total of 42 studies and 9834 CEA testing outcomes during follow-up [5]. Results point toward that CEA did not effectively detect treatable recurrences at an early stage. Besides, the low reliability of positive predictive value may lead to the high false positive rate. Therefore, considerations of factors and diseases known to influence CEA results and identification whether the increase in CEA is part of a fluctuating pattern or if it rises progressively are necessary.

FDG-PET and FDG-PET/CT are widely used for diagnosis, staging, evaluating therapeutic response, and detecting recurrence of a wide variety of cancers, including CRC. Many studies have demonstrated the value of PET in the detection of CRC recurrence in the post-operative patients with rising CEA [6–8]. Sometimes, the full-body scan of FDG-PET may detect occult synchronous or metachronous tumors. Focal FDG uptake within the thyroid gland can be associated with malignancy, most commonly papillary thyroid carcinoma [9]. Chen et al. had described thyroid incidentaloma identified by FDG-PET occurred with a frequency of 1.2% in 4803 physical check-up examinees, and 14% were proven to be malignant (all papillary carcinomas) [10]. Therefore, a hypermetabolic nodule in the thyroid gland could be either benign or malignant etiology, and further work-up with an ultrasound, FNA, or excisional biopsy is necessary.

The ultrasound-guided FNA of thyroid is a safe, inexpensive, minimally invasive procedure for determining thyroid malignancy and is an integral part of thyroid nodule evaluation. Limitations of FNA are related to the skill of the operator and the expertise of the cytopathologist. In a review of seven large series totaling 18,183 thyroid FNAs, Gharib et al. found the sensitivity of FNA for diagnosis of thyroid cancer ranges from 65 to 98% (mean, 83%), specificity ranges from 72 to 100% (mean, 92%), false negative rate ranges from 1 to 11% (mean 5%), and false positive rate ranges from 1 to 8% (mean 2.9%) [11]. According to the 2015 American Thyroid Association’s guidelines [12], repeat FNAC should be undertaken for the initial non-diagnostic cytology result of thyroid nodule in order to reduce the risk of false negative results.

MTC is a rare neuroendocrine malignancy arising from calcitonin-secreting parafollicular C cells in the thyroid gland. It accounts for approximately 3 to 10% of all thyroid neoplasms [13]. Reported sensitivity of FNA for diagnosis of MTC ranges from 53 to 89%, which is less than that of all thyroid cancer. On the other hand, serum calcitonin has an excellent sensitivity in diagnosing MTC from 98 to 99% [14, 15]. Serum CEA is another useful biomarker for MTC, especially in patients with poorly differentiated or metastatic MTC who may not have elevated serum calcitonin level [14]. Nevertheless, CEA level does not have the specificity of calcitonin for MTC. Calcitonin is a hormone produced and secreted by thyroid C cells specifically. Therefore, serum calcitonin levels can be determined as a highly specific biomarker for the early diagnosis of MTC [16]. Overall, FDG-PET/CT is less sensitive in MTC patients with de-differentiated tumors, whereas it is more sensitive in patients with high serum calcitonin (> 1000 ng/L) and short calcitonin doubling times (< 24 months) [17].

The patient’s history of resected CRC with high-serum CEA levels made us focus on searching recurrence or metastasis of CRC and overlook other disease causing elevated CEA level. If we checked his serum calcitonin while FDG-PET revealing hypermetabolic thyroid nodule or performed repeat FNAC in the next 3 months after initial negative cytology result, the delay in MTC diagnosis and treatment would have been avoided, and the disease might not spread to the cervical lymph nodes.

## Conclusion

FDG-PET/CT has emerged as a powerful imaging tool for the detection of the recurrence of colorectal cancers with rising serum CEA levels. The full-body scan of PET/CT may detect occult tumors that result in CEA elevation, such as MTC. This case reminds us that focal FDG uptake in the thyroid gland with elevated CEA level can be associated with MTC. Moreover, FNA has a higher false negative rate in detecting MTC than other forms of thyroid cancer. In this case, repeat FNA and measure of serum calcitonin could be most helpful in early detection of MTC.

## Abbreviations

CEA: Carcinoembryonic antigen
CRC: Colorectal cancer
CT: Computed tomography
FDG-PET: Fluorodeoxyglucose-positron emission tomography
FNA: Fine needle aspiration
maxSUV: Maximum standardized uptake value
MTC: Medullary thyroid cancer
